# Can large doses of glucocorticoids lead to Perthes? a case report and review of the literature

**DOI:** 10.1186/s12887-021-02755-4

**Published:** 2021-08-12

**Authors:** Guoming Chen, Tengyu Chen, Peng Zhang, Zhaoping Zhang, Ruilan Huang, Tao Chen, Wei He, Haibin Wang, Chi Zhou

**Affiliations:** 1 Guangzhou University of Chinese Medicine, Guangzhou, China; 2 First Affiliated Hospital of Guangzhou University of Chinese Medicine, Guangzhou , China

**Keywords:** Perthes disease, Glucocorticoids, Femoral head necrosis

## Abstract

**Background:**

Perthes disease (Legg-Calvé-Perthes, LCP) is a self-limited and non-systemic disease occurring in the femoral heads of children, which is mainly manifested as an ischemic necrosis of the femoral head epiphysis, leading to subchondral ossification injury of the femoral head.

**Case presentation:**

Here we report a case of 11-year-old child with long-term use of high-dose glucocorticoids. With MRI examination finding the epiphyseal necrosis of right humeral head, femur and tibia, and X-ray examination finding bilateral femoral head necrosis, the child was diagnosed as Perthes disease based on his clinical and imaging data.

**Conclusions:**

Long-term and high-dose glucocorticoids may be one of the causes of Perthes disease.

## Introduction

Necrosis of the femoral head in children, also known as Perthes disease (Legg-Calvé-Perthes, LCP), is a self-limited and non-systemic disease occurring in the femoral heads of children, which is mainly manifested as an ischemic necrosis of the femoral osteoepiphysis leading to subchondral ossification injury of the femoral head [1]. The clinical manifestations are mainly claudication and limitation of the affected hip joint movement.

Currently, it is generally believed that Perthes disease is a multifactorial disease caused by a combination of genetic and environmental factors. Genetic factors that mainly affect the sensitivity of the femoral heads to blood supply disruption and the tendency to thrombosis, and environmental factors that lead to trauma, such as excessive activities brought by childhood hyperkinetic syndrome, as well as maternal smoking and passive smoking, contribute to the disease. The tendency to thrombosis is one of the possible causes of Perthes disease that disputes mainly focus on, and some studies have demonstrated that the disease is associated with abnormalities in blood coagulation factors [2–10], whereas others do not [11–16]. The deficiency of protein C has been considered a possible etiological factor of Perthes disease [17, 18]. However, the meta-analysis of Matos M.A. et al. shows that there is insufficient evidence to prove that protein C is related to the pathogenesis of Perthes disease [19].

In addition, a growing body of research has revealed some other causes of Perthes disease, including repeated minor trauma [20, 21], vascular occlusion [22], bone growth stagnation [23, 24], abnormal insulin-like growth factor-1 pathway [25, 26], maternal smoking [27], secondhand smoke exposure [28–31], type II collagen mutation [32, 33], and low blood manganese level [34], but there’s not enough evidence to support them, and some studies have even questioned these views [35–38]. In recent years, the association between endothelial cells and Perthes disease has also been proposed [39, 40], but further studies are needed to prove it. In terms of its relationship with other diseases, Perthes disease has been reported to be associated with congenital malformation [41], fibrinolytic system disease [42], transient synovitis [43] and osteochondritis [44], which has been preliminarily recognized. Long-term use of glucocorticoids in large doses is associated with necrosis of the femoral head in adults [45–47], which is the most common and important risk factor that results in 10-30 % of necrosis of the femoral head in adults [48]. However, the relationship between glucocorticoids and necrosis of the femoral head in children is rarely documented in literature. At present, there is no research on the association between Perthes disease and the use of glucocorticoids throughout the world. This paper presents a case of the association between necrosis of the femoral head in children and the use of glucocorticoids, which would provide a new possibility for the causes of Perthes disease, so as to supplement the etiology and pathophysiology of this disease.

## Case presentation

### Patient Information and Clinical Findings

**Fig. 1:**
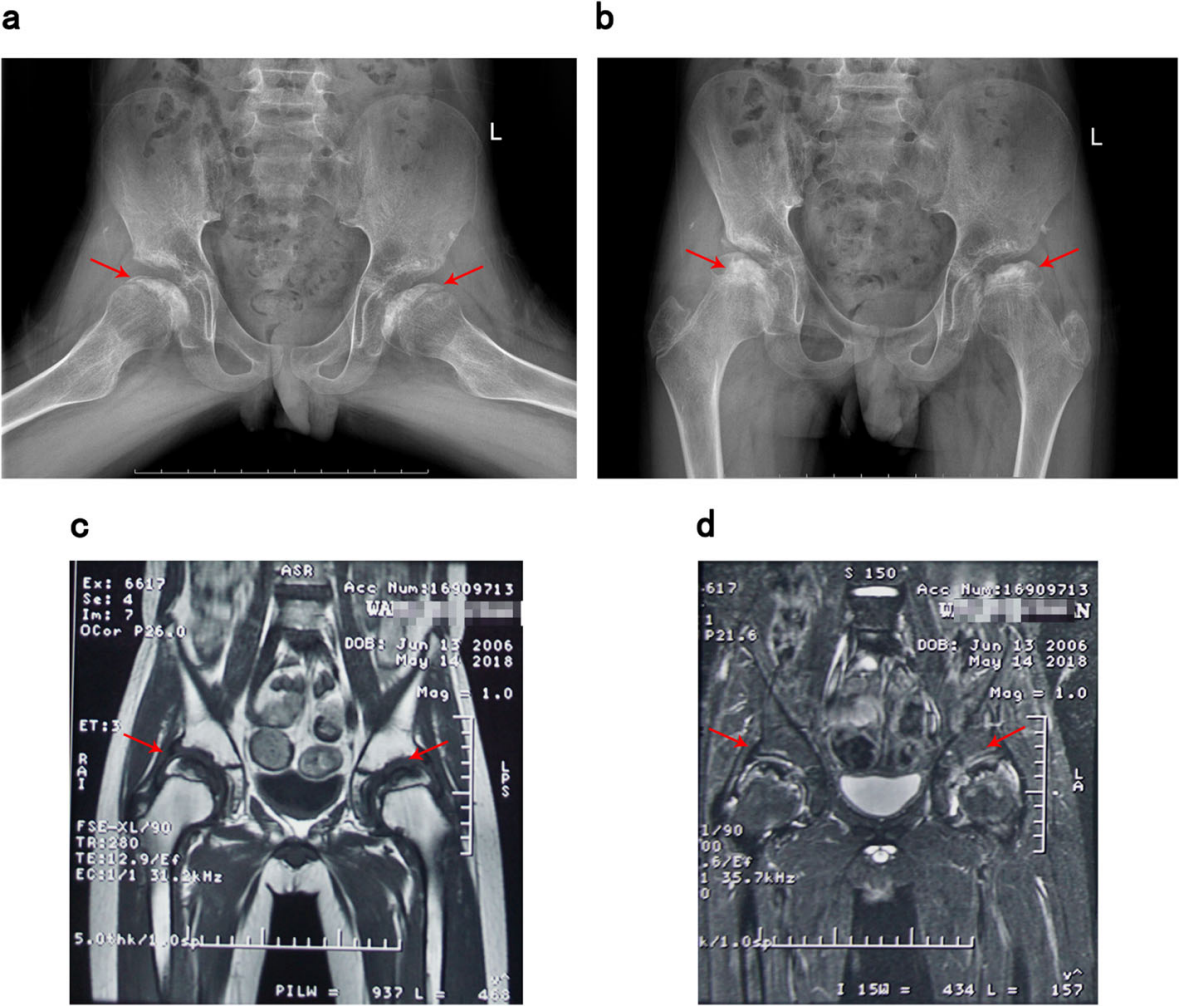
Patient’s bilateral hip X-ray and magnetic resonance imaging (**a-b**: hip X-ray imaging; **c-d**: hip magnetic resonance imaging. The arrows point out pathological structures in examinations, indicating bilateral necrosis of the femoral osteoepiphysis)

Wang, a 6-year-old boy, was hospitalized with “a week of limb aches after activities” in 2013, and was comprehensively diagnosed with “aplastic anemia” and then treated with cyclosporine (50 mg, bid). After genetic compatibility test, he was found to match his sister’s human leukocyte antigen (HLA) 10/10, so the allogeneic hematopoietic stem cell transplantation was successfully performed on June 2nd, 2016 (9 years old). The implantation rate was examined to be 85 % using fluorescence in situ hybridization (FISH) at 3 months after transplantation. He began to take methylprednisolone tablets (12 mg, bid), cyclosporin tablets (75 mg, bid) and mycophenolate mofetil tablets (250 mg, bid) after transplantation. Methylprednisolone tablets started to decrease after maintaining the dose for about half a year. In January 2017, the dosage of methylprednisolone tablets was reduced to 8 mg, bid, and then 4 mg, bid in February, and stopped in March. He persistently took cyclosporine (75 mg, bid) and mycophenolate mofetil tablets (250 mg, qd) since the transplantation. He didn’t take tacrolimus capsules (2.5 mg, tid), sirolimus tablets (1 mg, qd) and calcium carbonate D3 (1 tablet, qd) until rejection occurred in November 2017. In January 2018, he was diagnosed with “keratitis” due to photophobia and hyperemia in his left eye without obvious cause. Cytomegalovirus viral load DNA copy number was tested and in normal range (< 500 IU/ml). He received the therapy of ganciclovir (150 mg, bid), methylprednisolone (2 mg, bid) and cyclosporine (25 mg, qd) for the consideration of viral infection and chronic graft versus host disease (GVHD) after transplantation. Three months later, due to the pain and discomfort of both hips, he underwent bilateral hip X-ray and magnetic resonance examinations at the local hospital, which showed bilateral necrosis of the femoral osteoepiphysis (Fig. 1).

**Fig. 2:**
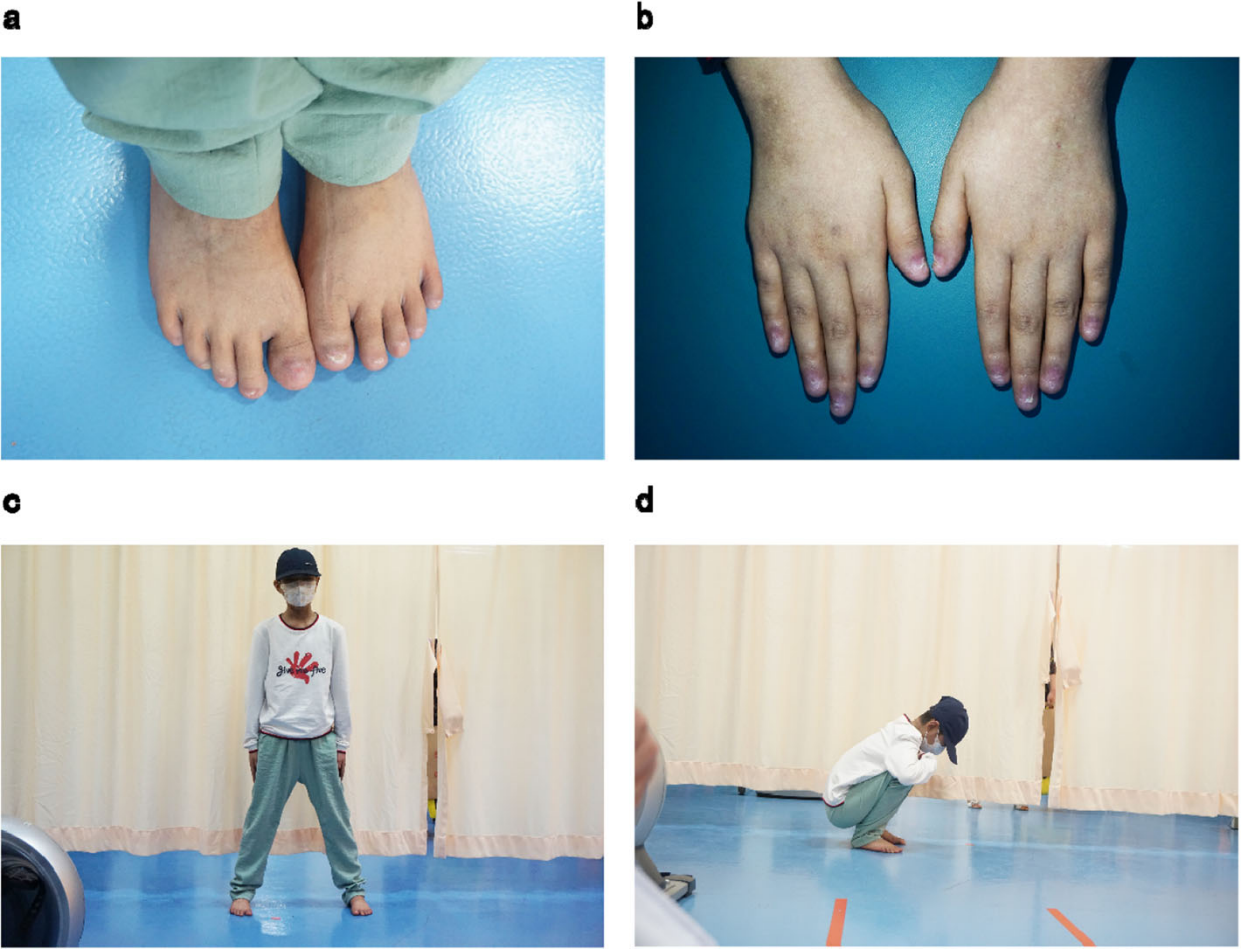
Patient’s manifestation of extremities (**a-b**: soften and lost fingernails and toes; **c-d**: The patient could stand and squat naturally. The joint pathologies did not influence patient’s articular function generally)

**Fig. 3:**
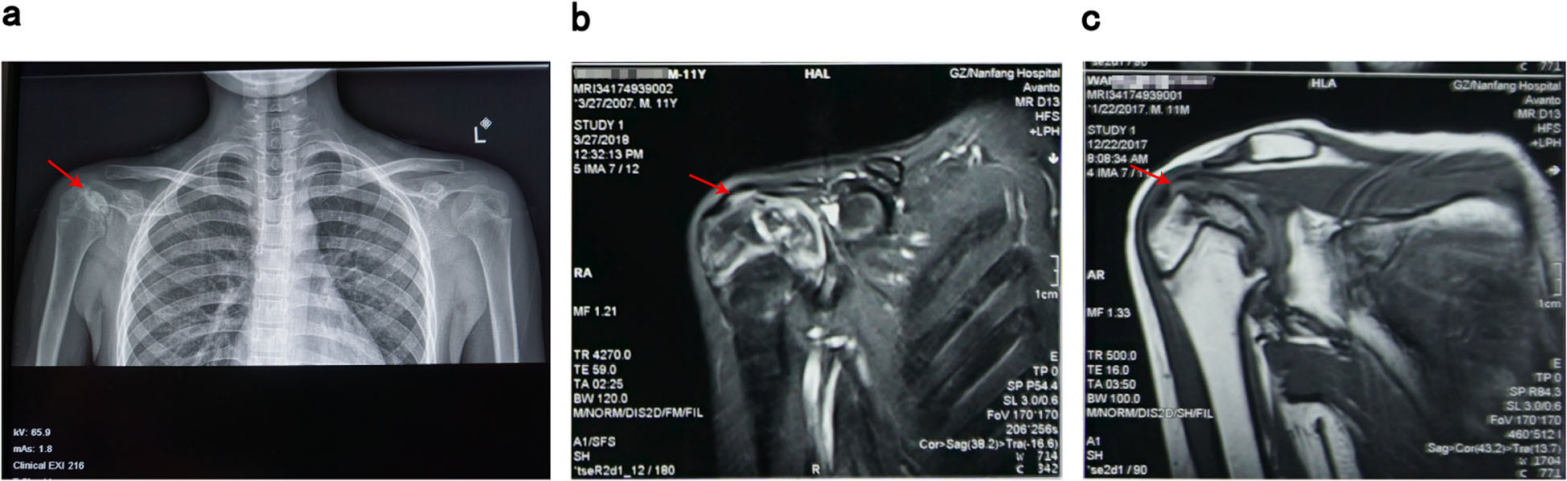
X-ray imaging (**a**) and magnetic resonance imaging (**b, c**) results of right shoulder joint (The arrows point out pathological structures in examinations, indicating necrosis of the right shoulder osteoepiphysis)

**Fig. 4:**
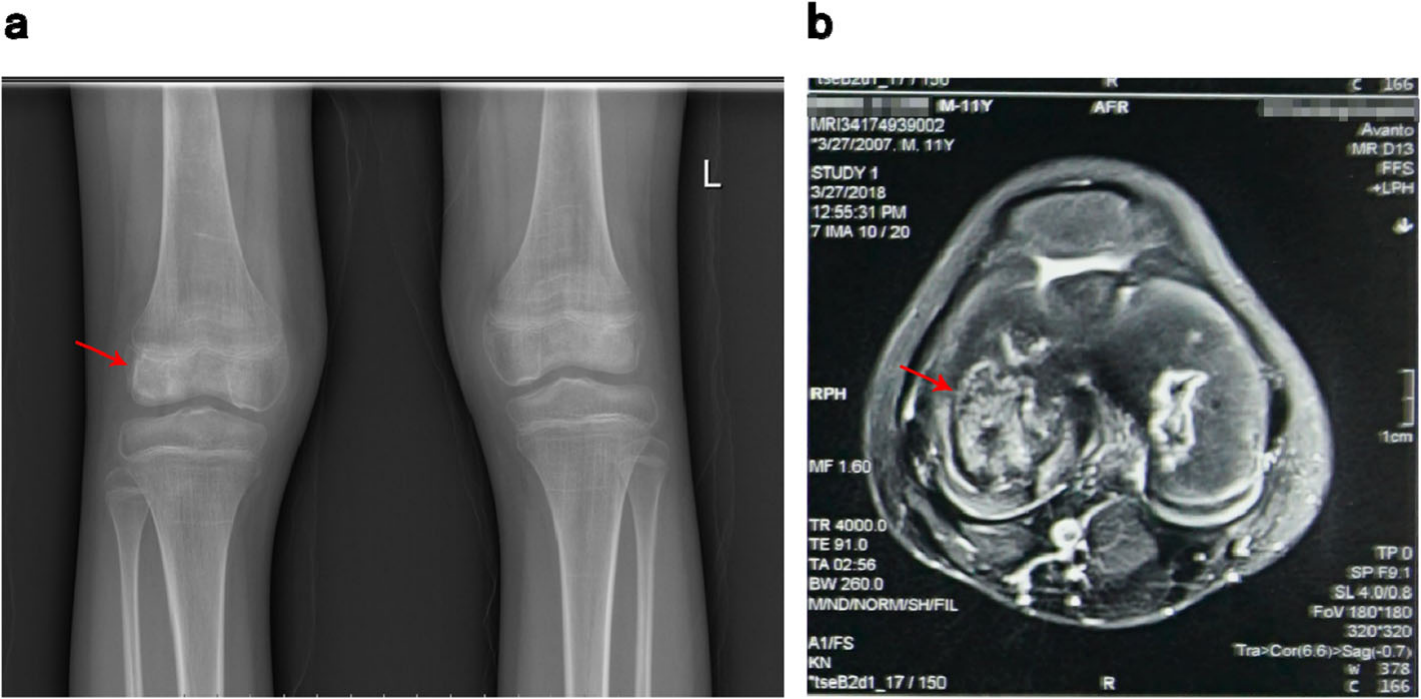
X-ray scan (**a**) and magnetic resonance imaging report (**b**) of knee joints (The arrows point out pathological structures in examinations, indicating necrosis of the right knee osteoepiphysis)

Subsequently, he was hospitalized again with “keratitis”, “aplastic anemia after hematopoietic stem cell transplantation”, and “bilateral necrosis of the femoral osteoepiphysis”. During his stay in the hospital, the drug concentrations of cyclosporine were monitored as CsA 175.00ng/ml and CsA 102.10ng/ml respectively on April 3rd and 9th, 2018, which normal level ranges from 100 to 200ng/ml. After admission to the hospital, ganciclovir (150 mg, bid), methylprednisolone (2 mg, bid) and cyclosporine (75 mg, bid) were given to alleviate graft-versus-host reaction, and then his visual acuity gradually improved. Methylprednisolone was discontinued after one month, and he was discharged on April 29th after his condition improved. After discharge, he continued taking the post-transplant medication. On May 22nd, he was hospitalized again with “two days of sore throat” with a large area of ulcer in his oral cavity. After treated with rituximab (5 mg, qw, 4 times in total), he continued to receive ganciclovir (150 mg, bid) antiviral treatment, and then his oral ulcers gradually improved. On May 26th, He developed the symptom of macroscopic hematuria, and urine routine examination showed positive urinary protein (1+) and positive urine occult blood (3+), while other indexes were normal. Blood analysis was reviewed on May 28th, and tacrolimus drug concentration was monitored as FK 506 11.60ng/ml (normal range: 5-10ng/ml), and sirolimus dose was adjusted at the same time of excluding infection. Blood routine test was reviewed on June 3rd, and he was treated with rituximab again. The treatment went on smoothly and the symptom of macroscopic hematuria was better than before. He was discharged on June 4th and continued to take the post-transplant medication. After discharge, he still felt painful in joints and could not walk easily. Worried about the deterioration of his condition, he was hospitalized in the First Affiliated Hospital of Guangzhou University of Chinese Medicine on June 19th. He complained of pain in movements of the hips, knees and ankles at admission, which was relieved after rest. After physical examination, he was found to have the following signs: multiple pigmentation spots throughout the body, keratitis, declining vision, softening of nails and toenails, limited rotation of both hips, double knee joint internal and external roll test (+), limited dorsal extension of ankle joints (Fig. 2). X-ray examinations of the shoulders, hips, knees and ankles and magnetic resonance examinations of the shoulders, knees and ankles were performed subsequently (Figs. 3 and 4), indicating epiphyseal necrosis of bilateral humeral heads and femoral heads, talus osteonecrosis, and femoral condyle bone infarction. Suffering from the disease at the age of 9, the 11-year-old child’s weight is stable at about 30 kg now, which is in line with the normal weight, indicating that there is no overweight and malnutrition for him. Since his transplantation in 2016, he began to take a large amount of glucocorticoids and anti-rejection drugs for a long period of time. During the past two years, every time the child took glucocorticoids, he was accompanied by pain in the hips, knees and ankles, which was relieved after the drug withdrawal. According to the analysis of his concrete condition, it is suspected that there could be a certain timeliness between necrosis of the femoral head and taking large doses of glucocorticoids (see Table 1).

**Table 1:** Therapeutic timeline of the patient

Date	Major symptoms	Therapy
2nd Jun 2016 (9 years old)	Limb aches	Allogeneic hematopoietic stem cell transplantation; adding methylprednisolone tablets (12 mg, bid), cyclosporin (75 mg, bid) and mycophenolate mofetil (250 mg, bid) orally
Jan 2017 (10 years old)	Limb aches (alleviated)	Methylprednisolone (reducing dose to 8 mg, bid), cyclosporin (75 mg, bid) and mycophenolate mofetil (250 mg, bid) orally
Feb 2017	Limb aches (alleviated)	Methylprednisolone (reducing dose to 4 mg, bid), cyclosporin (75 mg, bid) and mycophenolate mofetil (250 mg, qd) orally
Mar 2017	No obvious symptoms	Cyclosporin (75 mg, bid) and mycophenolate mofetil (250 mg, qd) orally
Nov 2017	Photophobia and hyperemia in patient’s left eye	Tacrolimus capsules (2.5 mg, tid), sirolimus tablets (1 mg, qd) as well as calcium carbonate and vitamin D3 chewable tablet (300 mg, qd) orally
Jan 2018 (11 years old)	Photophobia and hyperemia in patient’s left eye (worsened)	Ganciclovir (150 mg, bid), methylprednisolone (2 mg, bid) and cyclosporine (25 mg, qd) orally
Apr 2018	Photophobia and hyperemia; Pain in hips (bilateral necrosis of the femoral osteoepiphysis was diagnosed)	Ganciclovir (150 mg, bid), methylprednisolone (2 mg, bid) and cyclosporine (increasing dosage to 75 mg, bid) orally
May 2018	Photophobia and hyperemia (alleviated); Pain in hips (alleviated)	Methylprednisolone was discontinued; tacrolimus capsules (2.5 mg, tid), sirolimus tablets (1 mg, qd) and cyclosporine (75 mg, bid) orally
22nd May 2018	Sore throat; oral ulcer; pain in hips, knees and ankles	Tacrolimus capsules (2.5 mg, tid), sirolimus tablets (1 mg, qd), cyclosporine (75 mg, bid) and ganciclovir (150 mg, bid) orally; rituximab (5 mg, qw, the first time) subcutaneously
26th May 2018	Sore throat; oral ulcer; hematuria; pain in hips, knees and ankles	Tacrolimus capsules (2.5 mg, tid), sirolimus tablets (increasing dosage to 2 mg, qd), cyclosporine (75 mg, bid) and ganciclovir (150 mg, bid) orally
29th May 2018	Sore throat (alleviated); oral ulcer(alleviated); hematuria; pain in hips, knees and ankles	Tacrolimus capsules (2.5 mg, tid), sirolimus tablets (increasing dosage to 2 mg, qd), cyclosporine (75 mg, bid) and ganciclovir (150 mg, bid) orally; rituximab (5 mg, qw, the second time) subcutaneously
3rd Jun 2018	Sore throat (alleviated); oral ulcer(alleviated); hematuria (alleviated); pain in hips, knees and ankles	Tacrolimus capsules (2.5 mg, tid), sirolimus tablets (increasing dosage to 2 mg, qd) and cyclosporine (75 mg, bid) orally; rituximab (5 mg, qw, the third time) subcutaneously
10th Jun 2018	Pain in hips, knees and ankles	Tacrolimus capsules (2.5 mg, tid), sirolimus tablets (increasing dosage to 2 mg, qd) and cyclosporine (75 mg, bid) orally; rituximab (5 mg, qw, the fourth time) subcutaneously
19th Jun 2018	Pain in hips, knees and ankles; vision loss	Tacrolimus capsules (2.5 mg, tid), sirolimus tablets (increasing dosage to 2 mg, qd) and cyclosporine (75 mg, bid) orally

### Diagnostic Assessment

It has been reported in the literature that there are four distinct pathological stages in Caterall staging, herring typing, and the pathological course of Perthes disease: necrotic stage, cataclastic stage, repair stage, and plastic stage. Based on the imaging data and condition of this case, we comprehensively judged that the patient’s condition was in the second pathological stage of Perthes disease, the later stage of fragmentation, with necrotic bone absorption, new bone formation, mild collapses of the femoral heads, complete lateral column, and less smooth articular surface. In order to restore the normal mechanical properties of the femoral heads, the necrotic fracture must be absorbed, but rapid reabsorption could lead to collapse.

## Discussion and conclusions

Although increasing studies on the etiology or pathogenesis of Perthes disease have been performed for the past several decades, the causes of Perthes disease still remain unclear. In this case, with MRI examination finding the epiphyseal necrosis of right humeral head, femur and tibia, and X-ray examination finding bilateral femoral head necrosis, the child was diagnosed as Perthes disease based on his clinical and imaging data. And we suppose that long-term and high-dose glucocorticoids may be one of the causes of Perthes disease. It is generally recognized that excessive glucocorticoids can cause femoral head necrosis in adults, but regarding the use of glucocorticoids causing Perthes Disease, this case report may be the first to put forward this view. The child in this case can be diagnosed as Perthes Disease, but further discussion is needed about/on whether it is caused by drugs, especially glucocorticoids. Through the analysis of the following aspects, it could be concluded that the main cause of Perthes disease for the child in this case is the long-term and high-dose use of glucocorticoids.

The use of glucocorticoids in this case is characterized by long term and high dose. With respect to the medical history, the first time the child took glucocorticoids was at the age of 9 after transplantation in June 2016. At the beginning, methylprednisolone tablets (12 mg, bid) were used. It maintained for about half a year, began to be reduced to 8 mg from January 2017, halved in February and stopped in March 2017. The dose of glucocorticoids used during this period is moderate for adults but is already too high for children to be ignored. After his hospitalization in March 2018, methylprednisolone tablets began to be reduced to half a tablet, twice a day, but it was discontinued after short-term use because of the poor efficacy. From the transplantation in 2016 to 2018, the child underwent the treatment of high-dose glucocorticoids intermittently, and then aseptic necrosis was found in his right femoral epiphysis in March 2018. During the treatment, given that glucocorticoids may cause drug-induced femoral head necrosis not only in adults but in children, the clinicians were cautious to maintain the minimum effective dose, and adjusted the dosage in time to avoid poor efficacy or adverse reactions.

During the past three years of treatment, the child could not identify glucocorticoid specifically amongst the classes of drugs he was on at different times. However, every time he underwent the treatment of glucocorticoids, he suffered from pain in the hips but got relief after the drug withdrawal, which could exclude the interference from psychological factors of the patient. Besides, his two bone mineral density CT examinations showed the possibility of osteoporosis. It follows that the use of glucocorticoids is certainly associated with femoral head lesions.

In this case, immunosuppressants (cyclosporine, sirolimus, etc.) are also used and therefore it is also necessary to consider whether necrosis of femoral heads is the adverse reaction of immunosuppressant instead of glucocorticoids. Aside from methylprednisolone tablets, cyclosporine is the longest-used drug for this patient, which can help prevent allograft rejection. However, the use of cyclosporine in this case is in conventional dose, and there have been no reports of cyclosporine causing femoral head lesions in adults or children. On the contrast, studies show that patients with immunosuppressants such as cyclosporine or tacrolimus, for example, had lower rates of hip osteonecrosis after renal transplantation [49]. Therefore, the use of cyclosporine may not cause Perthes disease in this case. The treatment with rituximab, sirolimus and ganciclovir in 2018 was just for a short period, and the right femoral head lesions have been found before using these drugs, so they are also not related to the cause of Perthes disease in this case.

Regardless of whether it is for adults or children, aplastic anemia itself does not cause femoral head necrosis. Aseptic necrosis of the femoral head complicated by adult aplastic anemia is reported to be actually caused by the use of glucocorticoids during treatment [50]. Additionally, no research has ever shown that aplastic anemia can cause Perthes disease.

In this case, the 11-year-old child got sick about 2 years ago and his body weight was stable at about 30 kg, namely at a normal level, indicating that he was not overweight or malnourished, and thus his Perthes disease might not have much to do with his own growth and development process.

As a result, the femoral head necrosis of the patient in this case is most likely to be resulted from the long-term and high-dose use of glucocorticoids. Through this case report and literature review, glucocorticoids may be considered as a risk factor for Perthes disease. Although there’re some limitations during the whole therapeutic procedure, such as inadequate amounts of calcium and vitamin D supplements given for osteoporosis, doctors had prescribed herbal medicine or other alternative medicine for management of it. It is hoped that more cases could be reported in the future and related experimental studies could be performed to further validate and renew this view.

## Data Availability

Not applicable.

## Abbreviations

FISH: Fluorescence in situ hybridization
HLA: Human leukocyte antigen
LCP: Legg-Calvé-Perthes
